# Case of a Steatomatous Tumor under the Tongue

**Published:** 1826-05

**Authors:** John Bacot

**Affiliations:** Surgeon to the St. George's and St. James's Dispensary.


ART. VI.—*Case of a Steatomatous Tumor under the Tongue.* By JOHN BACOT, Esq. Surgeon to the St. George's and St. James's Dispensary.

THOMAS WHITEHEAD, aged about forty years, residing at No. 15, Crown-court, Brewer-street, was admitted under my care at the St. George's and St. James's Dispensary, on the 20th of March. The account which this man gave of his complaint was as follows:—Between six and seven years ago, he perceived a small swelling, about the size of a pea, under the integuments immediately beneath the chin, to which, as it produced neither pain nor inconvenience, he paid no attention: it, however, continued gradually to increase in size, and about a year ago its growth became more rapid, extending upwards within the mouth, until at length it encroached so much upon that cavity as to render deglutition difficult, and to impede his speech. So much, indeed, was the articulation interrupted, that it required some attention to make out the history which the man detailed. Upon looking into the mouth, the cause of the impediment of speech was sufficiently obvious: the tongue was not only greatly enlarged, but the tumor had pushed its way beneath that organ, so as completely to invert its usual position,—the inferior surface being turned upwards almost in contact with the roof of the mouth, and the apex forced backwards and a little to one side, in fact, nearly touching the left tonsil. That portion of the tumor which was situated below the chin was about the size of a pullet's egg, and appeared to contain some fluid, though the sense of fluctuation was not very distinct. It seemed to have no attachment to the larynx, nor to any of the important parts in the neighbourhood; and, on pressing the tumor upwards, the tongue was forced against the palate, so that it would have been easy to have produced suffocation by continuing the pressure. The man was unable, of course, to masticate or swallow solid food, and he was deprived of the sense of taste almost entirely. Excepting those inconveniences, however, the disease was not attended with any painful sensation.

In order to remedy this poor man's miserable situation, I proposed to make an incision in the direction of the tumor under the chin, believing it to be encysted, and probably filled with steatomatous matter; and then to puncture the cyst or lay it open, as might appear most advisable. This plan meeting with the approval of my colleague, Mr. Broughton, with his assistance, and in presence of the pupils, on the 23d of March, I made a vertical incision in the direction above mentioned, pursuing the dissection through the muscles. Finding that the sense of fluctuation was now much more distinct, though the cyst was situated more deeply than I had anticipated, I finished the operation by pushing an hydroçele trochar into the tumor. On withdrawing this, a small quantity of steatomatous matter escaped through the canula, but it was too thick and tenacious to flow out with great rapidity. The man had become very faint, and being unwilling to enlarge the opening if I could possibly avoid it, it was determined to retain the canula in the wound, through which the contents of the tumor continued to drain; the enlargement of the tongue having already decreased in some degree.

The next day, however, the canula having been permitted to escape from the wound, I was sent for in great haste. The swelling, both externally as well as within the mouth, appeared to be larger than before the operation, so that the patient was unable to speak at all, and appeared to be threatened with immediate suffocation: I therefore passed a probe into the opening made the day before, and then, with a common probe-pointed bistoury, enlarged the opening, so as to permit the passage of the canula of a large-sized trochar. A great quantity of cheesy matter now escaped, though mingled with fragments so thick and tenacious as to block up the canula completely, and to require the probe or director to be passed into the opening from time to time, in order to clear it out.

During the three following days, the tumor went on decreasing rapidly.

On Monday, the 27th March, Whitehead was enabled to swallow with more ease than for many months previously; and on that evening, whilst in the act of drinking, a great quantity of the same kind of matter suddenly gushed forth from the orifice, which was kept open either by a canula or director, which the man was enabled to pass into the cavity himself.

In the course of the following week, the tongue (which, though enlarged, preserved a healthy appearance,) began to resume its natural situation; so that yesterday, the 7th of April, the man was enabled to protrude it from the mouth, as usual. No vestiges of the tumor remain, excepting that the tongue yet appears to be too bulky at its lower surface, arising, most probably, from the long continuance of the disease.

It should be mentioned that, on the day succeeding the operation, Whitehead complained of much pain in the larynx, which was completely relieved by the application of leeches.

I have chiefly been induced to relate this case, because it appears to be somewhat uncommon. I do by no means wish it to be understood that the mode of operating which I adopted was the best that might have been pursued, because it is evident that, had a freer incision been made in the first instance, or a larger-sized trochar been employed, much of the embarrassment that afterwards was met with would have been avoided; but, as the situation of the tumor, as well as the appearances it produced, were unusual, the relation of this case may, perhaps, be useful to those who encounter a similar one; and this I conceive to be the principal, if not the only, benefit to be derived from the publication of solitary cases.

*South Audley-street; April 10th,* 1826.

